# Hearing intervention for decreasing risk of developing dementia in elders with mild cognitive impairment: study protocol of a multicenter randomized controlled trial for Chinese Hearing Solution for Improvement of Cognition in Elders (CHOICE)

**DOI:** 10.1186/s13063-023-07813-z

**Published:** 2023-11-28

**Authors:** Ying Chen, Lei Guan, Jie Chen, Kun Han, Qiongfei Yu, Jin Zhou, Xue Wang, Yunqian Ma, Xiangyu Ji, Zhonglu Zhao, Qiyue Shen, Anxian Wang, Mengping Wang, Jin Li, Jiali Yu, Yiwen Zhang, Sijia Xu, Jie Liu, Wen Lu, Bin Ye, Yuan Fang, Haixia Hu, Haibo Shi, Mingliang Xiang, Xia Li, Yun Li, Hao Wu

**Affiliations:** 1 Department of Otolaryngology-Head and Neck Surgery, Shanghai Ninth People’s Hospital, Shanghai Jiao Tong University School of Medicine, Shanghai, China; 2 Shanghai Key Laboratory of Translational Medicine On Ear and Nose Diseases (14DZ2260300), Ear Institute, Shanghai Jiao Tong University School of Medicine, Shanghai, China; 3 Department of Otolaryngology-Head and Neck Surgery, Shanghai Sixth People’s Hospital, Shanghai Jiao Tong University School of Medicine, Shanghai, China; 4 Department of Otolaryngology & Head and Neck Surgery, Ruijin Hospital, Shanghai Jiao Tong University School of Medicine, Shanghai, China; 5 Department of Geriatric Psychiatry, Shanghai Mental Health Center, Shanghai Jiao Tong University School of Medicine, Shanghai, China

**Keywords:** ARHL, Cognition, Hearing aids fitting, RCT, MCI

## Abstract

**Background:**

Age-related hearing loss (ARHL) signifies the bilateral, symmetrical, sensorineural hearing loss that commonly occurs in elderly individuals. Several studies have suggested a higher risk of dementia among patients diagnosed with ARHL. Although the precise causal association between ARHL and cognitive decline remains unclear, ARHL has been recognized as one of the most significant factors that can be modified to reduce the risk of developing dementia potentially. Mild cognitive impairment (MCI) typically serves as the initial stage in the transition from normal cognitive function to dementia. Consequently, the objective of our randomized controlled trial (RCT) is to further investigate whether the use of hearing aids can enhance cognitive function in older adults diagnosed with ARHL and MCI.

**Methods and design:**

This study is a parallel-arm, randomized controlled trial conducted at multiple centers in Shanghai, China. We aim to enlist a total of 688 older adults (age ≥ 60) diagnosed with moderate-to-severe ARHL and MCI from our four research centers. Participants will be assigned randomly to either the hearing aid fitting group or the health education group using block randomization with varying block sizes. Audiometry, cognitive function assessments, and other relevant data will be collected at baseline, as well as at 6, 12, and 24 months post-intervention by audiologists and trained researchers. The primary outcome of our study is the rate of progression to dementia among the two groups of participants. Additionally, various evaluations will be conducted to measure hearing improvement and changes in cognitive function. Apart from the final study results, we also plan to conduct an interim analysis using data from 12-month follow-up.

**Discussion:**

In recent years, there has been a notable lack of randomized controlled trials (RCTs) investigating the possible causal relationship between hearing fitting and the improvement of cognitive function. Our findings may demonstrate that hearing rehabilitation can be a valuable tool in managing ARHL and preventing cognitive decline, which will contribute to the development of a comprehensive framework for the prevention and control of cognitive decline.

**Trial registration:**

Chinese Clinical Trial Registry chictr.org.cn ChiCTR2000036139. Registered on 21 August 2020.

**Supplementary Information:**

The online version contains supplementary material available at 10.1186/s13063-023-07813-z.

## Background

Age-related hearing loss, also known as presbycusis, is a complex syndrome caused by the cumulative effects of aging on the auditory system, characterized by age-related, progressive, bilaterally symmetrical, and sensorineural hearing loss, which usually occurs at high frequencies in older adults firstly [1]. According to the Global Burden of Disease (GBD) Study in 2019, hearing loss has been the third leading cause of global years lived with disabilities among people aged 50 to 69, which became the primary cause among individuals aged 70 and above [2]. And through the data analysis and calculation, ARHL patients have increased from 751 million in 1999 to 1457 million in 2019, accounting for the most percentage of people with hearing loss [3]. The mechanism of ARHL is not clear at present [4], which considered to be a multi-factorial illness with potential risk factors including aging [5], sex [6], environment (noise exposure [7, 8], ototoxic drugs [9]), lifestyle (smoking [10], drinking [11]), comorbidities (diabetes [10, 11]), and genetic predisposition [1, 7, 12, 13].

ARHL induces a huge financial burden, which brings additional outlays of 1 trillion dollars a year [14]. However, there are no effective medicines or surgeries for ARHL until now, while the common intervention is wearing hearing aids [15]. Worse still, in the USA, the hearing aid intervention rate for individuals aged 50 and older was 14.2%, and the rate for individuals aged 65 and older was 6.5% in China [16, 17].

It has been recognized for decades that older adults with hearing loss had a higher risk of dementia [18, 19], in which cognitive decline interferes with the independent daily life of patients in memory, language, problem-solving, and other cognitive kills [20, 21]. Some hypotheses such as the cognitive load hypothesis, common cause hypothesis, and cascade hypothesis suggest the possible relationship between hearing loss and cognitive decline [22]. In recent years, researchers have found that hearing loss is an independent risk factor for accelerating the process of cognitive decline through cohort studies [23–26]. And the Lancet Commissions in 2020 pointed out that hearing loss is the first risk factor which can be preventable for Alzheimer’s disease, which population attributable fraction (PAF) accounted for about 8% of all of the 40% modifiable factors [27].

Nonetheless, the causality between ARHL and cognitive decline is not clear now [28]. According to a recent meta-analysis, the use of hearing aids may be linked to long-term cognitive benefits for older adults [29]. Mosnier compared the cognitive function between profoundly deaf older adults with or without cochlear implantation fitting and found that it was beneficial to the improvement of cognitive function [30]. Zhu found that patients aged 40–69 who have hearing loss without hearing intervention are at a heightened risk of developing all-cause dementia (HR 1.42 [95%CI 1.28–1.57]), while those with hearing loss who use hearing AIDS are not at an increased risk (HR 1.04 [0.98–1.10]) by using data from the UK Biobank [31]. However, in an observational study of 666 community-dwelling older adults with hearing impairment, there were no significant differences between hearing aid users and non-users in cognitive function, social engagement, or mental health outcomes during follow-up interval [32]. Another 6-month follow-up study found that hearing aids did not result in significant improvement in cognitive function among individuals with cognitive impairment and dementia [33]. Therefore, despite the challenges in studying the long-term cognitive effects of hearing intervention, we still need further studies, especially randomized controlled trials like the “ACHIEVE” project initiated by Frank Lin [34], to assess the potential benefits of hearing intervention on cognitive function.

MCI, occurring in the early process of cognitive development from normal to dementia, usually refers to a cognitive disorder which is significantly more severe than cognitive decline in normal aging, while patients retain most of the ability to perform independent activities of daily living [21, 35]. Its prevalence in individuals aged over 60 is reported to be 15.8% [36]. Patients with MCI will have a higher risk of dementia, and even if they return to normal cognition, the risk of subsequent cognitive deterioration is still higher than that of normal people who have never had MCI [37]. Many clinical researchers devote to delay the development of dementia in the pre-clinical stage, which makes MCI become the research hotspot in the dementia field [38, 39].

Since we pay more attention to their connection, the hearing intervention has been considered to be a potential therapy for cognitive decline [19]. Considering the few compelling evidence regarding the extent to which hearing aids play a role in cognition improvement among elders with moderate-to-severe ARHL and MCI, we designed this multicenter, randomized, controlled trial with two parallel groups. The CHOICE project, meaning Chinese Hearing Solution for Improvement of Cognitive in Elders conducted by Shanghai Ninth People’s Hospital, intends to provide the fundamental therapeutic strategy for ARHL management using hearing aids by establishing a cohort of ARHL patients (CHOICE-Cohort) and perform an RCT study (CHOICE-RCT).

## Methods/design

### Study design and settings

This multicenter, parallel-arm randomized controlled trial (RCT) study will be conducted by four tertiary referral clinics (Hospital of the Shanghai Jiao Tong University School of Medicine, China): Ninth People’s Hospital, Sixth People’s Hospital, Ruijin Hospital, and Mental Health Center. The study protocol is drafted following the Standard Protocol Items Recommendations for Interventional Trials (SPIRIT) statement (see additional file 1 for the SPIRIT 2013 Checklist) [40].

**Fig. 1:**
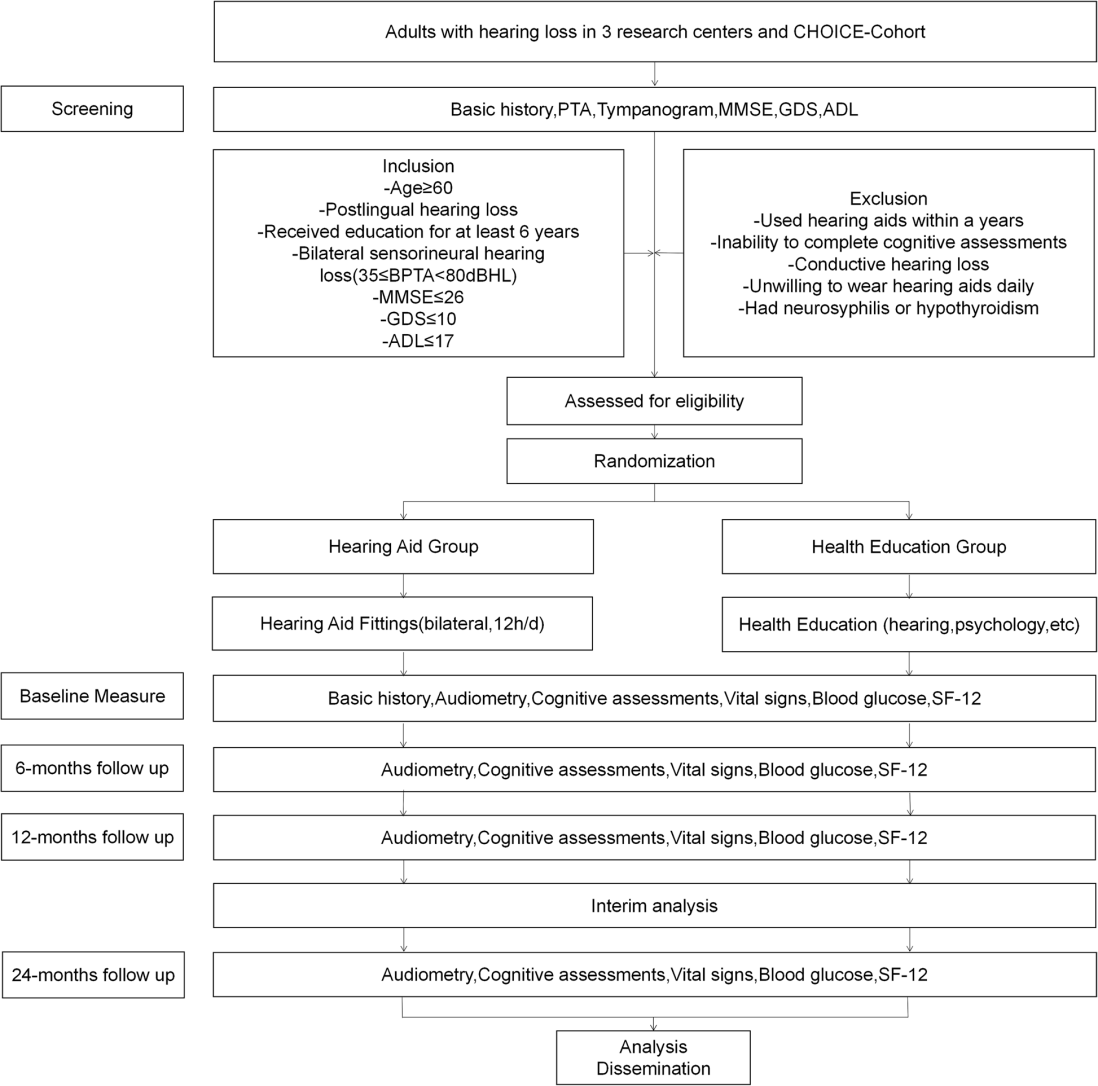
Flowchart for the study

Upon recruitment and screening, eligible participants will be randomly assigned to either the hearing aid fitting group or the health education group in a 1:1 ratio. These participants will then receive different interventions and undergo evaluations throughout the entire duration of the trial (see Fig. 1 for a flowchart of the study).

Baseline data will be collected at the start of the trial, followed by follow-up assessments at 6, 12, and 24 months after the interventions. These assessments will evaluate the primary outcome, which is the percentage of participants who develop dementia. Secondary outcomes, such as auditory and cognitive function evaluations, will also be measured in both groups.

By following this study design, we aim to compare the effectiveness of hearing aid fitting versus health education interventions in preventing the development of dementia and improving auditory and cognitive function. The participation of multiple centers will enhance the generalizability and robustness of our findings.

### Participants and recruitments

The study will include a diverse population, consisting of individuals seeking assistance for age-related hearing loss (ARHL) at the outpatient clinics of our study centers, those who have been informed about the study through online advertisements, and participants who are already enrolled in the cohort study of the CHOICE project. Since our investigation focuses on the development of dementia, which predominantly occurs in the late stages of life, we will specifically consider individuals who are aged 60 or older in order to ensure the external validity of our findings.

Upon providing signed informed consent, participants will be required to complete a questionnaire that covers their basic information, which is necessary for the research. Additionally, participants will undergo auditory tests and evaluations of cognitive function to fulfill the criteria established for the RCT study.

#### Inclusion criteria

- Age ≥ 60
- Postlingual hearing loss
- Received education for at least 6 years
- Sensorineural hearing loss
- 35 ≤ PTA (pure tone average at 0.5, 1, 2, 4 kHz) < 80 dB HL
- Mini-mental State Examination ≤ 26
- Geriatric Depression Scale ≤ 10
- Activity of Daily Living Scale ≤ 17

#### Exclusion criteria

- Used hearing aids within a year
- Inability to complete cognitive assessments
- Conductive hearing loss
- Unwilling to wear hearing aids daily
- Had neurosyphilis or hypothyroidism

### Randomization

Considering that the subjects in our experiment will be aware of whether they have received the hearing aid fitting, we will not be utilizing the double-blind method. In order to minimize potential bias and ensure a fair distribution of participants, we will randomly assign subjects from the outpatients and community cohort to either the intervention group or the control group in a 1:1 ratio. To prevent predictability, we will employ block randomization using variable block sizes [41]. This will involve generating randomization tables using Statistical Analysis System (SAS) software by our statistician. Cards will be prepared for the study, with allocation information concealed by a layer that matches the group sample size. Subjects will be recruited by our senior researchers in our project team, such as Dr. Hao Wu, Dr. Haibo Shi, Dr. Mingliang Xiang, Dr. Xia Li, Dr. Ying Chen, Dr. Wen Lu, Dr. Bin Ye, and Dr. Yuan Fang. They will enroll participants and obtain signed informed consent from all eligible participants. Participants will select their card in a predetermined order. And based on the allocation information of the card, the senior researchers will divide them into the hearing aid intervention group or the healthy education group. Once chosen, the card will not be returned.

### Intervention

After randomization, every participant allocated to the hearing aid fitting group will get the binaural and behind-the-ear hearing aids, Widex-Evoke E-FA100 & E-FP50, with a full set of auxiliary accessories, purchased by Ninth People’s Hospital and distributed to each sub-center. Our professional audiologist will make adjustments for patients until they can hear what others say with a few noisy environmental sounds, which will make participants willing to learn the usage of hearing aids and wear them in their daily lives. In addition, some of the subjects with a long history of hearing loss may lead to poor effect of hearing aids. We will provide some suggestions on hearing rehabilitation for them, including reading newspapers out and talking to their families frequently. Patients are required to wear hearing aids for more than 10 h per day. The time of wearing hearing aids will be recorded by a system; thus, we can remind subjects. Furthermore, participants will maintain their hearing aids if needed and make regular adjustments to their hearing aids with the help of an audiologist.

The patients in the health education group will receive periodic health education from researchers as part of the study, including valuable guidance on hearing health, such as the importance of noise reduction, avoidance of ototoxic drugs, and development of healthy ear care habits.

Furthermore, we will make it clear to the participants that those who are allocated to the intervention group will benefit from using hearing aids free of charge for the rest of their lives, while those who are assigned to the control group will receive an extra allowance at the end of the trial period. Besides, in the hearing aids group, we will use the system in hearing aids to record the average duration of hearing aid use, and for those who do not meet the requirement, we will take measures such as the commissioning of hearing aids and online supervision. All participants will get subsidies for the cost of transportation for every follow-up visit.

In the follow-up stage, the patients of the two groups will be recalled to study centers to perform assessments of auditory tests and cognitive evaluations at 6, 12, and 24 months after the intervention. Researchers will contact those who are near the date of the follow-up visit and make an appointment with them in advance.

Subjects are not allowed to participate in another trial which may influence the results during the study.

### Outcome measurements and follow-ups

The assessment of the following outcomes will take place during the screening, baseline measurements, and follow-up visits at 6, 12, and 24 months after the intervention (refer to Table 1). Our researchers will conduct all measurements using the same standardized procedures.

**Table 1:**
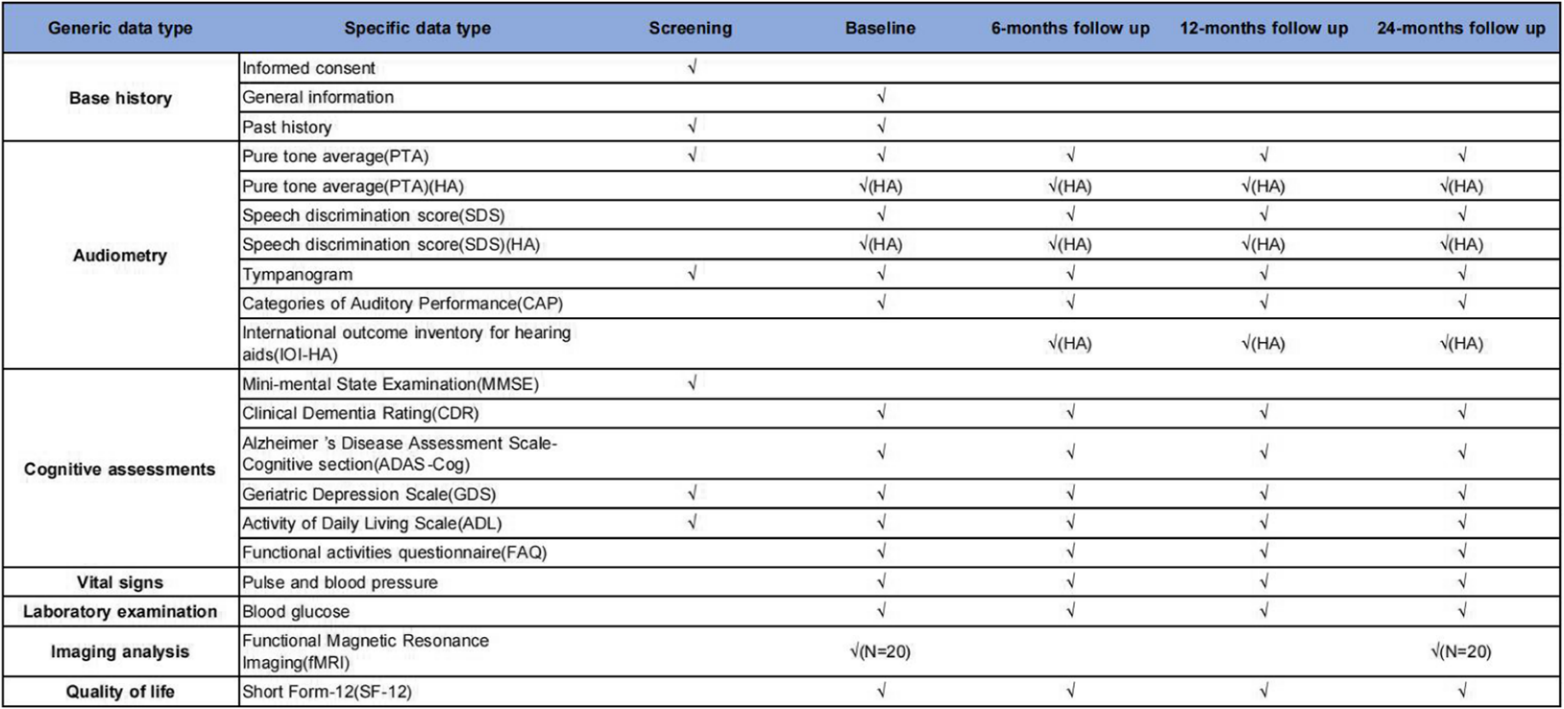
Outcomes and data collection in the study

“√(HA)” means that only participants in hearing aid fitting groups will perform the assessments, while “√” means both groups will perform them

#### Primary outcome measure

The primary outcome of the study is the rate of patients with ARHL and MCI progressing to dementia in two groups after different interventions.

#### Secondary outcome measures

##### General information

During the screening process, participants will be provided with a thorough understanding of our study and will be required to sign an informed consent form. Following this, our researchers will ask participants a series of questions regarding their educational background, hearing health, and whether they have used hearing aids within the past year. It is important to note that individuals with neurosyphilis or hypothyroidism will not meet the inclusion criteria, as these conditions may contribute to further cognitive decline in the patient [42, 43]. Once the screening is complete, eligible participants will be asked to complete a questionnaire specifically designed to collect basic information such as demographic characteristics, lifestyle factors, general health conditions, hearing-related symptoms, and any previous history of hearing disorders.

##### Audiological tests

Participants will undergo a series of audiological tests to evaluate the extent of their hearing loss. These tests will be conducted by audiologists from the hospitals at the study centers where participants have registered.

##### Pure tone audiometry

The main measurement is the pure tone audiometry. Our audiologists will evaluate the auditory threshold of the patients according to ISO 8253–1:2010 (en) [44] at a sound-treated booth. Patients will hear pure tones from small voice to loud at different frequencies through a headset. We intend to use the Astera (type1066) form NATUS, DNK, and Diagnostic Audiometer (AD229b) from INTERACOUSTICS, DNK to perform the audiogram as the evaluation results of the participants’ hearing level.

##### Tympanometry

Tympanometry is often used to assess the conditions of the middle ear, including the presence of fluid, changes in the internal structure, and the ear canal volume. It can be classified as type A, meaning the normal situation of the middle ear; type B, indicating that there is a significant abnormality in it; and type C, suggesting the negative pressure of the middle ear [45].

A listening test platform from INTERACOUSTICS A/S will be used to measure the tympanograms of both ears. Patients with type B will be ineligible for our study, which indicates the presence of fluid in the middle ear with the consequences of inflammation mainly, implying the secretory otitis media and potential conductive hearing loss [46].

##### Speech discrimination score (SDS)

Cognitive abilities, such as working memory and executive ability, play important roles in speech discrimination [47]. After the assessment of PTA, under the same initial environmental noise, the subjects will hear 10 monosyllables in the initial volume of their PTA + 20 dB HL at a sound-treated booth. We will record the percentage of correct monosyllables they retold as the SDS in the volume. By changing the signal-to-noise ratio (SNR), the maximum SDS among different SNRs will be confirmed when the participants’ SDS is up to 100% or the volume of monosyllables they heard reaches 90 dB HL. For the individuals, recovery will be considered if the maximum SDS increases by 10% or greater [48].

##### Categories of Auditory Performance (CAP)

CAP is usually used in assessing the auditory condition in patients with hearing loss and the process of recovery after hearing intervention [49]. The original CAP cannot describe the sophisticated listening skills of adults, so we are going to use an extended version of it [50], which consists of ten categories, graded from 0 “No awareness of environmental sounds” to 9 “Use of phone with unknown speaker in unpredictable context,” which will correspond to different auditory receptive abilities.

##### International outcome inventory for hearing aids (IOI-HA)

IOI-HA is an international standard self-report questionnaire developed to quantify the satisfaction of hearing aid users and the improvement of their lives by the application of hearing aids [51]. It consists of 7 questions, including the time of wearing hearing aids, the benefit and satisfaction of hearing aid applications, etc. Each question is ranging from the worst performance 1 to the best performance 5 [52].

##### Cognitive evaluations

Cognitive function encompasses various domains, including basic sensory and perceptual processes, as well as advanced executive function and cognitive control abilities [53]. To conduct a comprehensive evaluation of patients’ cognitive function, we plan to utilize a series of assessment scales. These questionnaires have previously been translated into Chinese and are commonly used in clinical settings. All evaluations of the patients’ cognitive will be performed at our study centers by our researchers and will not be disturbed by other things.

##### Mini-mental State Examination (MMSE)

MMSE consists of temporal and spatial localization, word retelling, calculation, language use, comprehension, and basic motor skills [54], which has been the most common method for decades to detect the presence of cognitive decline [55]. It will be performed in the screening phase of the study. And although hearing loss will not conduct a significantly additional impact on the scores of MMSE result [56], we will raise the volume of the conversation with participants properly, ensuring that participants will hear the evaluation contents correctly.

##### Clinical Dementia Rating (CDR)

CDR is widely used in the measurement of the patient’s cognitive impairment, which consists of evaluations in six domains (memory, orientation, judgment and problem-solving, community business, home and hobbies, personal care). The score of 0 in CDR represents normal, while 0.5 represents MCI or suspected dementia and 1, 2, and 3 represent mild, moderate, and severe dementia [57].

##### Alzheimer’s Disease Assessment Scale-Cognitive section (ADAS-Cog)

ADAS-Cog is usually performed to evaluate the cognitive and behavioral functions in patients impaired by Alzheimer’s disease (AD) and is considered to be the gold standard for evaluating the efficacy of dementia treatment [58]. The scale is made up of 11 subscales designed to assess a variety of cognitive abilities, including memory, speech, and practice, which are often thought to be the features in the development of AD [59]. In our study, we will select the Chinese translation version with 12 items and add the item of attention, which also passed the reliability and validity analysis [60].

The minimal clinically relevant change (MCRC) on the ADAS-Cog for patients with AD is usually among 3 to 5 points. 4 points are recommended by the FDA as the appropriate MCRC for clinical trials of patients [61].

##### Other evaluations

**Geriatric Depression Scale (GDS)**

Depression is usually suggested being a risk factor and a prodromal symptom of dementia [62]. So we will use the original GDS, a 30-item self-report scale, to assess the situation of depression in our subjects, which helps us distinguish it in MCI patients with more than 10 points [63, 64]. The scale represents features of depression in older adults in both emotional (e.g., sadness, crying, loneliness) and cognitive areas (e.g., hopelessness, helplessness, guilt) [65].

**Activity of Daily Living Scale (ADL)**

ADL-14 consists of 14 questions, involving the whole set of behaviors of people in daily life, devised for evaluating the activity functional state of individuals systematically and personalized by asking their family numbers. The higher score on ADL-14 indicates greater functional disability [66].

**Functional activities questionnaire (FAQ)**

FAQ is commonly used to assess the functional limitations in older adults [67]. The questionnaire contains 10 questions for evaluating complex cognitive functions and social activities. It consists of handling finances, purchasing daily necessities, preparing meals, the attention to events broadcast in the media, etc., ranging from 0 (independent) to 3 (dependent on others totally) in each content. A score of 9, meaning the subject is completely dependent on others in at least 3 domains, is suggested functional impairment in patients [68]. We will interview their family members to obtain the information.

**Short Form-12 (SF-12)**

The SF-12 is the shortened alternative to the SF-36, containing 12 subsets. It is often used to evaluate the living quality of participants with a focus of whole physical and mental health outcomes [69, 70].

##### Vital signs and blood glucose

According to the previous study, cumulative blood pressure is one of the risk factors in dementia with negative cognitive effects [71]. And high blood glucose is associated with the risk of unspecified dementia [72]. Therefore, we will measure the subjects’ pulse, blood pressure, and glucose to determine whether subjects have any underlying disease. Furthermore, these data will also suggest the physical states of patients to monitor whether serious adverse events occur.

### Adverse events

No foreseeable adverse events related to hearing aid fitting are expected to occur in the study. However, in the event of any unexpected adverse events, they will be documented and reported following the standard operating procedures set forth by the Ethics Committee of Shanghai Ninth People’s Hospital.

### Participants’ withdrawal

Participants have the right to withdraw from the study at any time without providing a reason. Any participants who are lost to follow-up will be recorded, and an intention-to-treat analysis will be performed exclusively for these individuals.

### Blinding

Blinding is not possible in this study due to the nature of the intervention for participants or researchers, but we intend to blind the data analyzers to reduce bias in the data analysis. Moreover, we will employ several strategies to mitigate bias from both researchers and participants. Firstly, prior to their involvement in the CHOICE project, researchers will receive standardized training to ensure consistent data collection and analysis. Additionally, we will utilize standardized measurement tools and quality control procedures to monitor data accuracy and reliability. Furthermore, we will ensure that participants in both the intervention and control groups are well-informed about the potential benefits and drawbacks of their respective treatments, aiming to minimize biases in their perceptions of therapeutic effects.

### Statistical analysis

#### Sample size

To detect the clinically relevant difference in the dementia rate after the hearing aid fitting compared with the health education group, the sample size of this study is calculated as follows. Our pilot study results showed that 3 of 30 patients with ARHL and MCI wearing hearing aids developed dementia within a year. Literature reported that 18% of the elderly with MCI progressed to dementia every year [73]. In conclusion, by using PASS software (Vision 15), *α* was set at 0.05, 1 – *β* = 0.8, and hearing aid fitting and health education group were set at 1:1; thus, 292 cases in each group were calculated. Considering the 15% of drop-out rate, we designed 344 cases in each group, and a total of 688 cases. Shanghai Ninth People’s Hospital will be responsible for 516 cases, Shanghai Sixth People’s Hospital for 103 cases, and Shanghai Ruijin Hospital for 69 cases. Furthermore, Shanghai Mental Health Center will provide training about cognitive evaluations for our researchers and recommend eligible patients for our study.

#### Data collection

All research centers will use the paper Case Report Form designed for the study to record the original data. Then, they will be input into the Shen-kang Clinical Research Integration Platform system by trained researchers from Shanghai Ninth People’s Hospital. The database is password-protected, which can only be accessed by specified researchers. All changes made in the database will be logged. The final data set will be only available to authors for research.

The research centers will utilize the designated paper Case Report Form (CRF), specifically designed for this study, to record the original data. Subsequently, trained researchers from Shanghai Ninth People’s Hospital will input the data into the Shen-kang Clinical Research Integration Platform system. Access to the database is protected by a password and limited to authorized researchers. Any modifications made within the database will be meticulously logged. The final dataset will solely be accessible to the authors for research purposes.

#### Statistical methods

The mean and standard deviations, or the median and quartiles, will be used to describe the baseline features of both the hearing aid fitting group and the health education group, depending on the normality of the data. The unpaired *t*-test will be used to calculate the differences between the control and intervention groups. For within-group comparisons, paired *t*-tests will be used for continuous measures. Non-parametric tests will be used to compare data that do not follow a normal distribution. The differences in outcomes between groups will be determined by calculating the means and 95% confidence intervals. A *P* value < 0.05 will be considered statistically significant. Additionally, mixed effects models, COX regression, and multifactorial linear regression will be utilized to further explore the association between hearing aid fittings and cognitive improvement.

Intention-to-treat analysis will be conducted on all subjects enrolled in the group. Additionally, a per-protocol analysis will be performed on subjects with high compliance, specifically those who have worn hearing aids for the duration required by the experiment. To handle missing data, multiple imputation will be utilized. Complete case analyses will be conducted as a sensitivity analysis. It is important to note that all analyses will be performed on an intention-to-treat basis.

We will perform an interim analysis when all subjects complete the 12-month follow-up visits using the baseline data and follow-up data collected at 6 and 12 months after the interventions, and if the results of the interim analysis are positive, we will consider terminating the study.

### Quality control

Researchers, including audiologists, cognitive evaluators, and data collectors, will be required to ensure full compliance with specifications and pass the consistency test during the assessment process. Additionally, 5% of the subjects will be randomly selected for audio recording of the entire cognitive evaluation process, which will be submitted to the Shanghai Mental Health Center for quality control.

An external data monitoring committee will monitor the study. The committee will be independent of the sponsor and researcher in the study and have no competing interests. The committee will have an audiologist, a psychiatrist, and a statistician, who will review the provisional data when the completion of data collection for the first year. The implementation of projects will be tracked and reviewed by the Ethics Committee of Ninth of Shanghai Ninth People’s Hospital every year.

### Ethics and dissemination

All subjects will participate in the study voluntarily, and the senior researchers in our project team, such as Dr. Hao Wu, Dr. Haibo Shi, Dr. Mingliang Xiang, Dr. Xia Li, Dr. Ying Chen, Dr. Wen Lu, Dr. Bin Ye, Dr. Yuan Fang, will obtain their written consent forms from each individual prior to their enrolment in the group. It is expected that both groups of subjects will derive benefits from the study. Additionally, we will inform all participants about any new information that may influence their willingness to participate in the experiment. The trial will adhere to the criteria and principles outlined in the Declaration of Helsinki and has received approval from the Ethics Committee of Shanghai Ninth People’s Hospital (project ID: ChiCTR2000036139) [74]. We will also be subject to an annual ethical review. Furthermore, we have registered the trial on chictr.org.cn. Any modifications to the study will be promptly communicated to the Ethics Committee of the Ninth Hospital and will be updated on the registration website once approval is obtained.

The entirety of the research-related data, including original data and records, quality control files, and software used for data storage and follow-up analysis, will be limited to access by the study team only. The statistical analysis and processing of the collected data will be conducted exclusively by designated researchers. The identity and contact information of all participants will be strictly confidential. Prior to performing statistical analysis, the data will be anonymized. Ultimately, the research findings will be published in reputable scientific journals.

## Discussion

One focus of ARHL studies is the causal relationship between hearing loss and cognitive decline. In recent years, research has shown that hearing loss is the most significant modifiable factor in preventing dementia. However, currently, there is limited research on hearing interventions that improve the cognitive function of ARHL patients, particularly in randomized controlled trials. The scarcity of studies may be attributed to the prolonged development process of dementia or the absence of clear evaluative indicators for cognitive function, which adds to the challenge of conducting RCT trials. The ACHIEVE project, the sole published large-scale randomized controlled trial, focused on whether the use of hearing aids reduces the incidence of dementia among cognitively normal individuals over a 3-year follow-up period and yielded negative findings [75].

According to previous research, about 16% of older adults have MCI, and individuals with MCI have a higher likelihood of progressing to dementia at a faster rate compared to those with normal cognitive function. Furthermore, cognitive impairment is often accompanied by hearing loss in older adults, with nearly half experiencing moderate hearing loss or worse [76]. Therefore, our intention is to specifically recruit patients with MCI for this study, which fits the purpose of the experiment and will help us obtain more evident results in the study. Additionally, to evaluate cognitive function, we will employ a variety of assessments. Each questionnaire will be weighted, and a final score will be calculated. This composite score is expected to serve as a more discernible and objective measure of participants’ overall cognitive ability.

In conclusion, in addition to monitoring the development of dementia, we intend to investigate the effectiveness of hearing intervention on various cognitive domains in patients with ARHL and MCI. We anticipate that hearing rehabilitation can be proven to be an effective therapeutic approach for cognitive recovery, thus enhancing our ability to prevent and manage ARHL.

## Time points/timeline, trial status, and publication plan

Recruitment for this randomized controlled trial (RCT) study commenced in 2021, with the first patient enrolled on September 28, 2021. Currently, subject enrollment is still in progress (November 2023). It is anticipated that the recruitment, screening, random allocation, and baseline evaluation of all participants will be finalized by 2023. Following the completion of the 1-year follow-up for all subjects, the gathered data will be utilized for an interim analysis.

Protocol Version 3: 18 November 2023.

## Fundings

The study is funded by the Clinical Research Plan of SHDC (SHDC2020CR1044B to HW), Natural Science Foundation of Shanghai (23ZR1437100 to YC), National Natural Science Foundation of China (82371141 to YC), and Biobank Project of Shanghai Ninth People’s Hospital, Shanghai Jiao Tong University School of Medicine (YBKA202205 to YC). All research funding for our study is handled by the research department of Ninth People’s Hospital, so we provide the contact information of the hospital research funding administrator, Ms. Qing Li, with the contact email kjc5325@vip.163.com. Moreover, in the study, the sponsor is not involved in the research design, data collection, data management, data analysis and interpretation, writing of the manuscript, and publication of the paper. They will not have ultimate authority over any of these activities.

### Supplementary Information

**Additional file 1. **SPIRIT 2013 Checklist: Recommended items to address in a clinical trial protocol and related documents.

## Data Availability

The datasets analyzed during the current study and statistical code will be available from the corresponding author on reasonable request in the future, as is the full protocol, too.

## Abbreviations

ADAS-Cog: Alzheimer’s Disease Assessment Scale-Cognitive section
ADL: Activity of Daily Living Scale
ARHL: Age-related hearing loss
CAP: Categories of Auditory Performance
CDR: Clinical Dementia Rating
CHOICE: Chinese Hearing Solution for Improvement of Cognition in Elders
FAQ: Functional activities questionnaire
GDS: Geriatric Depression Scale
IOI-HA: International Outcome Inventory for Hearing Aids
MCI: Mild cognitive impairment
MMSE: Mini-mental State Examination
SDS: Speech discrimination score
SF-12: Short Form-12
